# The comprehensive influence of critical thinking disposition and problem-solving ability on the labor literacy of adolescents

**DOI:** 10.3389/fpsyg.2026.1702960

**Published:** 2026-02-20

**Authors:** Fengying Wei, Caixia Tian, Tongfeng Li

**Affiliations:** 1 College of Education, Qinghai Normal University, Xining, China; 2 The College of Computer, Qinghai Normal University, Xining, China

**Keywords:** critical thinking disposition, labor literacy, problem-solving ability, relationship, teenagers

## Abstract

**Purpose:**

This study investigated the correlation between critical thinking disposition and labor literacy among Chinese adolescents and examined the mediating role of problem-solving ability. These findings aim to emphasize the importance of education in cultivating critical thinking and problem-solving skills among adolescents, improving the literacy of students’ labor and promoting their overall development.

**Method:**

A questionnaire survey was conducted among 2,268 junior and senior high school students, including the California Critical Thinking Disposition Inventory Test (CCTDI) (Revised Edition in China), the Problem-solving Evaluation Questionnaire (Revised Edition), and the Labor Literacy Assessment Questionnaire for Middle School Students.

**Results:**

(1) There are significant gender differences in the critical thinking disposition and labor literacy of teenagers (*p* < 0.05), while there are no significant differences in terms of place of residence (*p* > 0.05). There are significant differences in problem-solving ability and labor literacy between junior high school and senior high school stages (*p* < 0.05). (2) The critical thinking disposition, problem-solving ability, and labor literacy are positively correlated with each other in pairs. (3) The direct effect of the critical thinking disposition of teenagers on labor literacy is significant, with an effect size of 66.42%; the mediating effect of problem-solving ability on the relationship between critical thinking disposition and labor literacy is significant, with an effect size of 33.58%.

**Conclusion:**

This study confirms that the critical thinking disposition of teenagers not only directly positively influence their labor literacy, but also play a crucial mediating role by enhancing problem-solving abilities. This finding reveals the core mechanism of labor literacy cultivation: Labor education should aim to cultivate “thinking workers” - individuals who can actively analyze, judge, and innovatively solve complex real-world problems by applying critical thinking. Therefore, educational practice needs to systematically design labor tasks that integrate real problem scenarios, so as to simultaneously promote the coordinated development of critical thinking and labor literacy during the process of training students’ problem-solving abilities, thereby providing empirical evidence and implementation paths for building a high-quality labor education system in the new era.

## 1 Introduction

In the new era where globalization and technological revolution intertwine, international competition increasingly manifests as a competition of human resource quality. The United Nations Educational, Scientific and Cultural Organization (UNESCO) has listed “learning to know” and “learning to do” as the two main pillars of education. This indicates that critical thinking and labor literacy are important topics in the cultivation of talents in the 21st century, and they are the foundation for individuals to cope with future challenges and promote social innovation and development. In the face of many potential challenges brought about by social changes and technological revolution, the youth group may encounter problems such as value disconnection and weak practical ability. Against this backdrop, the educational value of labor literacy has been re-examined and elevated to the national strategic level. Labor education centered on labor literacy has become an important part of the education system in various countries. In recent years, China has explicitly proposed to incorporate labor education into the entire process of talent cultivation, aiming to build an education system that comprehensively nurtures morality, intelligence, physical fitness, aesthetics, and labor literacy. This measure is highly consistent with the international trend of viewing labor education from the perspectives of “holistic development” and “core competencies.”

Labor literacy is not merely physical activities or skill training; rather, it refers to the comprehensive qualities of correct values and key abilities that teenagers internalize through continuous and educational labor practices. It plays an irreplaceable foundational role in the growth of teenagers and is the cornerstone for their self-sufficiency, pursuit of happiness, and future success. Through labor, teenagers can most directly understand responsibility and shape sound values. At the same time, labor transforms abstract knowledge into problem-solving abilities and cultivates social responsibility and resilience through collaboration. The practical wisdom, innovative consciousness, and hands-on skills gained through “learning by doing” are beyond the reach of books and are the core foundation for their future adaptation to social changes and realization of personal value. According to Bronfenbrenner’s ecosystem theory, the formation of teenagers’ labor literacy is influenced by multiple levels of systems in interaction. In the immediate micro-system, school labor education, individual academic performance, structure (whether they are the only child), parents’ labor concepts and attitudes, etc., constitute the direct experiences for their development. Existing research (Wu et al., 2024; Wang and Song, 2024) has confirmed the significant role of these factors. In the outer macro-system, such as social culture and environment, they provide a time-oriented guidance for the development of labor literacy. Through dynamic interaction among these systems, the possibility of individual labor literacy development is jointly shaped.

Critical thinking, as an advanced cognitive ability, has increasingly become a cutting-edge literacy in the labor market (Penkauskienė et al., 2019). It is not only a tool for analyzing and evaluating information, but also the core for individuals to achieve career development, helping job seekers enhance vocational training, cultivate reflective attitudes, and develop core abilities for lifelong learning (Zhao et al., 2024). In the current challenging employment environment, teenagers should systematically cultivate critical thinking as early as possible to enhance their future core competitiveness. Dewey argued that true learning stems from experience and is achieved through “learning by doing”, unifying experience and action. When individuals face real problems, they need to use reflective thinking to actively explore and solve problems, thereby achieving experience transformation and knowledge construction. Dewey’s theory established the internal unity of thinking, action, and learning at the philosophical level, providing a framework for understanding the relationship between critical thinking, problem-solving, and labor literacy. Later scholars developed “reflective thinking,” such as scholars like Facione, through Delphi research, clearly defining this thinking as “critical thinking” and proposing a two-dimensional model, considering “cognitive skills” and “emotional tendencies” as the two major dimensions of critical thinking (Facione, 1990). Ennis further refined the composition of critical thinking dispositions, such as seeking clarity and maintaining openness, which determine the possibility of individuals’ continuous engagement in complex tasks (Ennis, 2011). Siegel, from the perspective of educational philosophy, argued that critical thinking as the core of rational autonomy is the prerequisite for individuals to make judgments and take actions in social and professional life (Siegel, 2013). These theoretical developments indicate that critical thinking dispositions are the key psychological mechanism that drives cognitive skills into practice and points toward labor participation. Numerous studies have also confirmed the significant value of critical thinking. As an advanced cognitive skill, it can enhance logical reasoning ability and problem-solving efficiency (Dwyer et al., 2014), and has a significant predictive effect on academic performance (Akpur, 2020) and scientific creativity (Qiang et al., 2020), etc. The critical thinking disposition dimension is directly related to deep learning and career development (Geng et al., 2024). Relevant surveys show that Chinese youth groups generally perform well in critical thinking dispositions (Gillet et al., 2017). Therefore, the cultivation of critical thinking has become a core issue for individual social adaptation and overall educational quality. More and more countries around the world have listed it as a key training target at all levels of education (Li et al., 2024).

Problem-solving ability is the ultimate manifestation of critical thinking in practical terms. It is a comprehensive ability of individuals to analyze and evaluate complex situations through critical thinking and ultimately achieve the expected goals. It is also a key ability for individuals to cope with complex challenges in the future society. The “General Problem Solver” model proposed by Newell and Simon regards problem-solving as an information processing process through heuristic strategies to search for paths, emphasizing the importance of cognitive structure and strategies (Newell and Simon, 1972). With further research, scholars have paid more attention to problem-solving in complex real-world situations. “Ill-structured” problems often lack clear goals, paths, and answers. Solving them requires the collaboration of critical thinking, domain knowledge, and metacognitive monitoring (Jonassen, 2000). This directly links problem-solving ability with higher-order thinking and practical literacy. These theories indicate that problem-solving ability is an integrated psychological trait that drives individuals to use critical thinking, mobilize resources, and achieve goals in complex and uncertain situations. It is the key for individuals to adapt to society and the core competitiveness for succeeding in future careers (Autor and Dorn, 2009), and can significantly enhance employees’ competitiveness and adaptability (Mainert et al., 2019). A large number of empirical studies support its close association with critical thinking: individuals with high levels of critical thinking have greater advantages in problem identification and solution, and the two are significantly positively correlated (Wang et al., 2024).

The cultivation of labor literacy is the core for strengthening labor education and achieving its educational goals. It is the basic standard for realizing the goals of labor education (Wu et al., 2024), and it is a key indicator for assessing whether an individual can achieve meaningful participation and development in the real working world. Critical thinking, as the cognitive foundation of labor literacy, has been elaborated in multiple dimensions. As Siegel argued, the rational autonomy cultivated by critical thinking enables workers to become active practitioners who can make prudent judgments and take on responsibilities (Siegel, 2013). Empirical research strongly supports this connection: Indrašienė et al. (2021) demonstrated that employees with a strong critical thinking disposition are better able to adapt to changing environments in the workplace and demonstrate a high sense of responsibility for work quality. Zhao (2012) macro analysis also pointed out that in the knowledge economy, critical thinking is the core cognitive capital for workers to achieve technological upgrading, role transformation, and lifelong learning. These studies confirm that individuals with good critical thinking skills can make better decisions in complex situations and enhance their overall employment competitiveness (Barton and McCully, 2007; Dwyer et al., 2014).

Problem-solving ability is the direct manifestation of labor literacy in complex situations. Globalization and technological progress have made labor tasks increasingly complex, requiring workers to be able to handle “ill-structured problems.” Therefore, cultivating problem-solving ability essentially involves forging the core resilience of workers to deal with uncertainties. In terms of teaching methods, problem-based learning (PBL) provides students with a practical field for connecting knowledge, thinking, and action by simulating challenges in real labor scenarios (Heong et al., 2020), which is an effective way to develop comprehensive labor literacy.

Labor literacy is a key indicator of an individual’s labor ability, and the critical thinking disposition is the necessary preparation for driving critical thinking activities. Together, these two elements constitute the core competencies necessary for adapting to future complex challenges. Existing research has begun to focus on the potential connections between cognitive abilities and labor literacy, but the internal mechanism of this relationship and its universality across different adolescent groups have not been fully explored. Specifically, critical thinking, as a core 21st-century skill, is believed to enhance an individual’s analytical and decision-making abilities in complex situations, thereby improving the quality and literacy of their labor practice. However, is this theoretical path robust in all adolescent groups? Students of different genders, from different urban and rural backgrounds, and at different educational stages may have systematic differences in thinking traits, ability development, and labor experiences. Will these differences significantly regulate the transformation process of “thinking - ability - literacy”? The answers to these questions not only concern the completeness of the theoretical model but also have direct guiding significance for achieving fair, effective, and precise labor education practices. This study aims to conduct an empirical investigation of the adolescent population using the questionnaire survey method, describe the current development status and characteristics of critical thinking disposition and labor literacy among adolescents, deeply analyze the influence path of critical thinking disposition on labor literacy, test whether and how problem-solving ability plays a role between the two, and thereby reveal the internal mechanism. It will also systematically examine the differences of this mechanism in different demographic groups and test the universality of the theoretical model, providing solid empirical evidence and theoretical support for the construction of a youth literacy cultivation model. Based on the above integrative theoretical framework, this study proposes the following core hypotheses:

Hypothesis 1: Critical thinking disposition is positively correlated with problem-solving ability.

Hypothesis 2: Critical thinking disposition is positively correlated with labor literacy.

Hypothesis 3: Problem-solving ability is positively correlated with labor literacy.

Hypothesis 4: Problem-solving ability acts as a mediating factor between critical thinking disposition and labor literacy.

**Figure 1:**
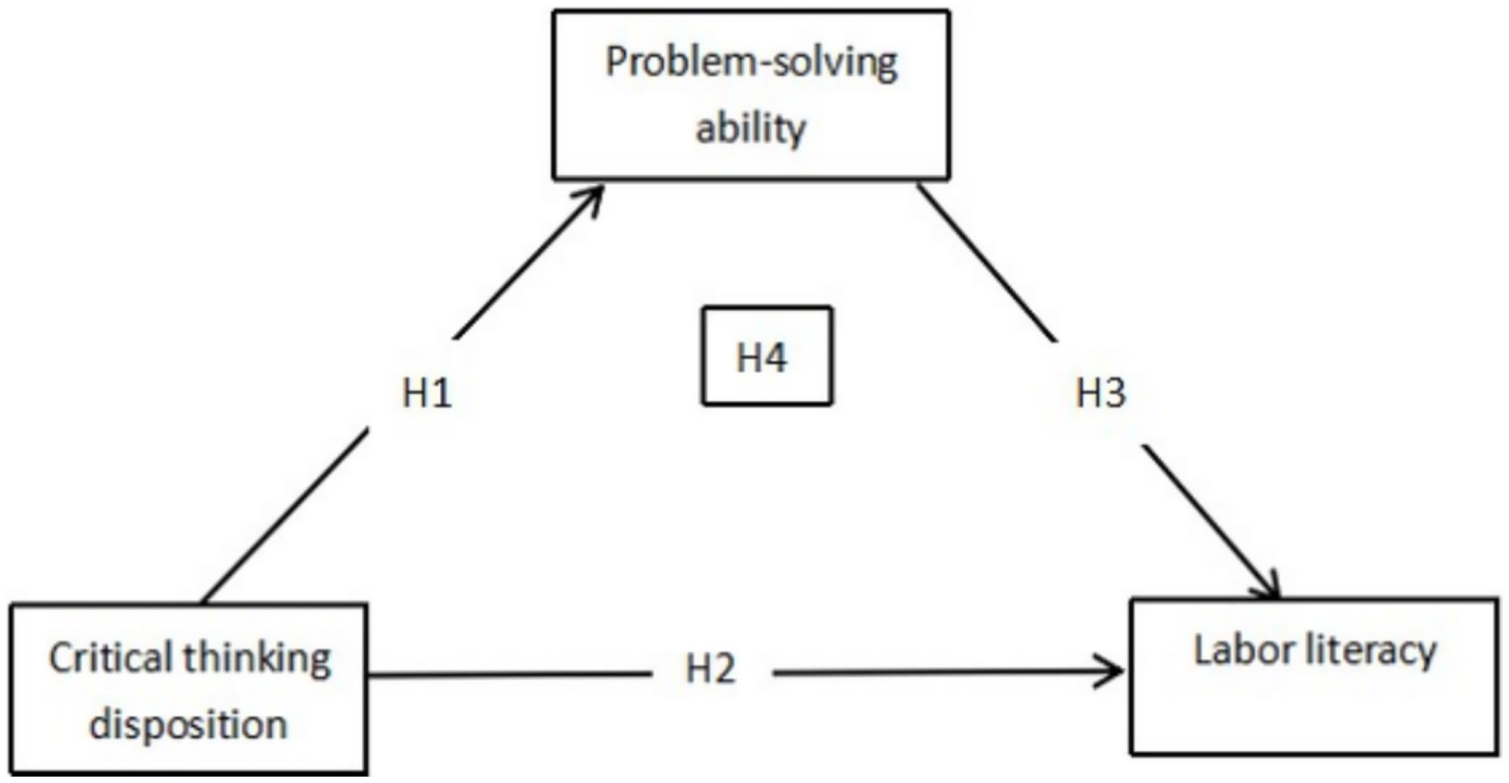
Conceptual architecture model.

The hypothesis model is shown in Figure 1.

## 2 Materials and methods

### 2.1 Participants

This study is based on the educational goal system for cultivating talents with all-round development in the five aspects of “morality, intelligence, physical fitness, aesthetics, and labor” during the basic education stage in China. The labor education goal under this system refers to “labor literacy,” which is completely different from the labor ability in the field of social economy. Therefore, this study does not involve concepts and fields related to social economy and the labor market. Two provinces, Qinghai and Hainan, were selected as samples. This was mainly based on the principle of theoretical sampling, according to the regional differences in China’s education development, to test the universality of the psychological mechanism. Although the social economic backgrounds of the two regions were slightly different, both carried out educational practices under the unified labor education training goals and evaluation standards. This enabled us to explore the internal development patterns of students across different contexts. By using a convenient sampling method, students from junior and senior high schools in several schools in Qinghai Province and Hainan Province were selected as the survey subjects. The survey period was from November 23, 2024 to June 11, 2025. To protect the subjects’ right to know, before filling out the questionnaire, the subjects were informed of the content to be filled out, and their consent was obtained. Then, 2,674 questionnaires were distributed through the Wen-juan-xing platform, and 2,268 valid questionnaires were recovered, with an effective recovery rate of 84.82%. Among them, there were 1,018 boys, accounting for 44.89%, and 1,250 girls, accounting for 55.11%.

### 2.2 Methods

#### 2.2.1 Problem-solving evaluation questionnaire

Problem-solving ability refers to the individual’s tendency to adopt a positive attitude, effectively apply methods and strategies when facing a problem situation, and demonstrate psychological qualities such as perseverance and confidence during the process. The study employed the “Problem-solving Evaluation Questionnaire” revised by Liu Youxia et al. in 2015. It used a 5-point Likert scale, with each item having 5 levels (1 = completely disagree, 2 = disagree, 3 = generally, 4 = agree, 5 = completely agree). There were a total of 31 items, among which 4 were open-ended questions, divided into “attitude dimension,” “method and strategy dimension,” and “quality dimension.” The higher the score, the stronger the problem-solving ability. The Cronbach’s *α* = 0.95, and the Cronbach’s α of each dimension were: 0.88, 0.76, and 0.94.

Attitude dimension: refers to the stable psychological tendency and reaction mode that an individual has toward others or things, including cognition, emotion, and intention. For example: I can persist for a long time to focus on solving a difficult problem.

Method and strategy dimension: refers to the tendency and ability of an individual to consciously invoke and effectively apply systematic and planned cognitive and behavioral steps in the process of solving problems. For example: I use knowledge, strategies, and tools from the subject field to solve problems.

Quality dimension: refers to the ability to appropriately and creatively solve problems and obtain reliable and excellent substantive results. For example: I can clearly list the key points of the complex problem to be solved.

#### 2.2.2 The critical thinking disposition inventory scale

The critical thinking disposition refers to the mental preparedness and habitual willingness of an individual to actively apply analytical, evaluative and reasoning skills in cognitive activities. It is specifically manifested as thinking traits such as inquiry, seeking truth, openness, systematicness and confidence toward things. The Critical Thinking Disposition Scale is a questionnaire revised from the California Critical Thinking Disposition Inventory test (CCTDI) by Chinese scholars Peng-Meici et al. based on the interpretation of localization. It uses the Likert 6-point scale (1 = very agree, 2 = generally agree, 3 = agree, 4 = disagree, 5 = generally disagree, 6 = very disagree), with a total of 70 items. Each scale consists of 10 questions, including 30 positive questions and 40 negative questions. It consists of 7 scales, measuring the 7 traits of critical thinking disposition, namely seeking truth, open-mindedness, analysis ability, systematic ability,critical thinking of self-confidence, intellectual curiosity and cognitive maturity. The total score ranges from 70 to 420. A score below 210 indicates a negative critical thinking disposition (low), a score between 211 and 279 indicates an unclear meaning (medium), and a score above 280 indicates a positive critical thinking disposition (high). The scores for each trait disposition range from 10 to 60, with scores below 30 indicating a negative trait manifestation (low), scores between 31 and 39 indicating an unclear meaning (medium), and scores above 40 indicating a positive trait manifestation (high). The Cronbach’s *α* = 0.78.

Seeking truth: Adopt a sincere and objective attitude toward seeking knowledge. Even if the answer found does not align with one’s original viewpoint, contradicts one’s beliefs, or affects one’s own interests, it is not worth caring about. For example: Even if there is evidence that contradicts my thoughts, I will still stick to my own ideas.

Open-mindedness: Adopt a tolerant attitude toward different opinions and guard against the possibility of personal bias. For example: Understanding others’ thoughts about things is important for me.

Analysis ability: Can identify the problem, understand the crux and predict the consequences with reasons and evidence. For example: When dealing with difficult problems, first clarify the crux of the problem.

Systematic ability: Organize and aimedly strive to handle problems. For example: I am good at planning a systematic plan to solve complex problems.

Critical thinking of self-confidence: Have confidence in one’s rational analysis ability. For example: I am satisfied with being able to come up with creative choices.

Intellectual curiosity: Be curious and passionate about knowledge, and try to learn and understand, even if the practical value of these knowledge is not directly obvious. For example: I like to figure out how things work.

Cognitive maturity: Make prudent judgments, or refrain from making judgments, or modify existing judgments. Be vigilant in accepting multiple problem-solving methods. Even in the absence of comprehensive knowledge, I can understand that even an expedient decision is sometimes necessary. For example: I am firmly convinced of what I believe.

#### 2.2.3 Labor literacy questionnaire

Labor literacy refers to the basic qualities that an individual acquires through active participation in labor education and labor activities, including labor concepts, labor abilities, labor habits, and labor qualities. The labor literacy assessment questionnaire for middle school students was compiled by Chinese scholars Wang Guang-qiang and others. It is divided into two dimensions: labor concepts and labor abilities, with a total of 9 items. The Likert 5-point scoring method is used (1 = very inconsistent, 2 = inconsistent, 3 = uncertain, 4 = consistent, 5 = very consistent),and the higher the score,the higher the student’s labor literacy. The Cronbach’s *α* = 0.97.

Labor concepts include labor identity (identifying and respecting the value and social relationships of the labor activities) labor attitude (respecting, recognizing, and voluntarily participating in labor activities), and labor values (the significant importance and role of labor in human life and social development). For example: I believe that labor plays an important role in the all-round development of people.

Labor abilities include labor knowledge application ability (understanding the concept and historical development of labor,and mastering and applying knowledge related to household labor and production labor), labor planning ability (planning for labor schemes,selection of materials and tools, etc.),and tool usage ability (being able to proficiently use various labor tools in different labor situations). For example: I understand and learn labor theoretical knowledge, and constantly innovate in the labor process.

#### 2.2.4 Data analysis

In this study, common method bias tests, descriptive statistics, correlation analysis, independent sample t-tests, analysis of variance and linear regression analysis were conducted using SPSS 27.0, Process v4.1 by Andrew F. Hayes to process and analyze the collected data. The mediating effect analysis was conducted using Hayes’ PROCESS v4.1, and the model was Model 4. Among them, M = problem-solving Ability (Mediator), X = critical thinking disposition (Independent Variable), Y = labor literacy (Dependent Variable), and the Bootstrap sample = 5,000.

#### 2.2.5 Data collection process

Before the data collection, all the students involved in the research were given a comprehensive explanation of the research purpose. Additionally, the participants were assured that the research would not have any physical or psychological effects. Furthermore, it was specifically stated that the research results would not affect school grades, and students’ names would not be collected. The data collection tools were collected from the students in their classroom environment through a network platform. Participation in the research was voluntary. On average, it took 32 min to complete each questionnaire (Table 1).

**Table 1:** Demographic data of the study group.

Demographic variable	Category	*n*	Percentage (%)
Gender	Male	1,018	44.89
	Female	1,250	55.11
Educational stage	Junior high school	1,679	74.03
	Senior high school	589	25.97
Place of household registration	Town	889	39.20
	Rural	1,379	60.80

## 3 Results

### 3.1 Common method bias test

The Harman univariate test extracted a total of 11 factors with eigenroots greater than 1. Among them,the first factor cumulatively explained 27.46% of the total variance, which was less than the critical value of 40%, indicating that there was no serious common method bias in this study. The inspection standard is *α* = 0.05.

### 3.2 Descriptive statistics and correlation analysis

The research focuses on the overall scores of the variables in the model and uses the average scores of the variables for Pearson correlation analysis. Descriptive statistics show (as shown in Table 2): that critical thinking disposition is positively correlated with problem-solving ability, problem-solving ability is positively correlated with labor literacy, and critical thinking disposition is positively correlated with labor literacy.

**Table 2:** Mean, standard deviation and correlation coefficient between variables.

Variables	*M ± SD*	1	2	3
1. Critical thinking disposition	236.76 ± 22.57	1		
2. Problem-solving ability	88.95 ± 20.58	0.436**	1	
3. Labor literacy	21.97 ± 8.09	0.373**	0.394**	1

*M* = Mean, SD = Standard deviation. ^∗∗^*p* < 0.01 (The same below).

### 3.3 Independent sample *t*-test and analysis of variance

#### 3.3.1 The test for differences in each variable across genders

In this study, an independent sample t-test was employed to analyze the gender of the participants. The results revealed significant differences in the critical thinking disposition and labor literacy of Chinese teenagers across genders. Specifically, boys scored significantly higher than girls in the dimensions of open-mindedness, analysis ability, intellectual curiosity, and cognitive maturity. In the critical thinking of self-confidence dimension, girls scored significantly higher than boys. In terms of labor literacy, boys also scored significantly higher than girls in the labor concept dimension (Table 3).

**Table 3:** Comparison of differences in each variable and its dimensions across genders (*N* = 2,268).

Variables	Male (*n* = 1,018) *M ± SD*	Female (*n* = 1,250) *M ± SD*	*t*
Critical thinking disposition	238.92 ± 21.54	235.00 ± 23.23	4.13***
Seeking truth	36.98 ± 9.61	36.25 ± 7.84	1.94
Open-mindedness	34.78 ± 5.25	33.60 ± 5.16	5.37***
Analysis ability	32.55 ± 5.34	32.01 ± 4.85	2.53*
Systematic ability	34.41 ± 4.62	34.24 ± 4.78	0.85
Critical thinking of self-confidence	31.65 ± 8.22	32.46 ± 7.00	−2.52*
Intellectual curiosity	31.56 ± 6.14	30.88 ± 5.96	2.65**
Cognitive maturity	37.00 ± 9.55	35.56 ± 7.80	3.86***
Problem-solving ability	88.54 ± 23.28	89.28 ± 18.08	−0.82
Attitude	25.84 ± 7.42	25.61 ± 5.97	0.80
Methods and strategies	25.40 ± 6.48	25.73 ± 5.31	−1.31
Quality	37.30 ± 10.76	37.93 ± 8.51	−1.52
Labor literacy	22.39 ± 8.83	21.63 ± 7.42	2.20*
Labor concept	9.83 ± 4.16	9.43 ± 3.50	2.50*
Labor ability	12.56 ± 4.88	12.20 ± 4.21	1.83

∗*P* < 0.05; ∗∗∗*P* < 0.001 (The same below).

#### 3.3.2 The difference test of each variable in terms of household registration

In terms of the place of household registration, only in the sub-dimensions of “analysis ability” and “intellectual curiosity” did the urban areas score significantly lower than the rural areas. There were no significant differences in other aspects (*p* > 0.05) (Table 4).

**Table 4:** Comparison of differences in each variable and its dimensions across household registration (*N* = 2,268).

Variables	Town (*n* = 889) *M ± SD*	Rural (*n* = 1,379) *M ± SD*	*t*
Critical thinking disposition	235.62 ± 22.70	237.50 ± 22.46	−1.94
Seeking truth	36.52 ± 8.55	36.61 ± 8.78	−0.23
Open-mindedness	33.93 ± 5.27	34.26 ± 5.21	−1.46
Analysis ability	31.98 ± 5.03	32.43 ± 5.11	−2.07*
Systematic ability	34.29 ± 4.59	34.33 ± 4.78	−0.19
Critical thinking of self-confidence	31.88 ± 7.46	32.24 ± 7.66	−1.10
Intellectual curiosity	30.77 ± 5.97	31.45 ± 6.09	−2.60*
Cognitive maturity	36.25 ± 8.41	36.18 ± 8.81	0.17
Problem-solving ability	88.86 ± 19.02	89.01 ± 21.53	−0.17
Attitude	25.75 ± 6.30	25.69 ± 6.89	0.23
Methods and strategies	25.70 ± 5.56	25.51 ± 6.05	0.78
Quality	37.40 ± 8.90	37.81 ± 10.01	−1.01
Labor literacy	21.83 ± 7.85	22.06 ± 8.24	−0.66
Labor concept	9.57 ± 3.71	9.63 ± 3.88	−0.36
Labor ability	12.26 ± 4.39	12.43 ± 4.61	−0.88

#### 3.3.3 The difference test of each variable in terms of educational stage

During the junior high school and senior high school stages, there were significant differences in the scores of problem-solving ability and labor literacy among teenagers (*p* < 0.05). Specifically, in terms of the total score of problem-solving ability and its dimensions of attitude, methods, and strategies, as well as the total score of labor literacy and its dimensions of labor concepts and labor abilities, junior high school students scored significantly higher than senior high school students. However, in the systematic thinking dimension of critical thinking disposition, junior high school students scored significantly lower than senior high school students (Table 5).

**Table 5:** Comparison of differences in each variable and its dimensions across educational stage (*N* = 2,268).

Variables	Junior high school (*n* = 1,679) *M ± SD*	Senior high school (*n* = 589) *M ± SD*	*t*
Critical thinking disposition	236.68 ± 24.02	236.98 ± 17.80	−0.32
Seeking truth	36.46 ± 9.45	36.90 ± 6.02	−1.30
Open-mindedness	34.25 ± 5.48	33.78 ± 4.44	2.07
Analysis ability	32.32 ± 5.40	32.05 ± 4.05	1.30
Systematic ability	34.08 ± 4.96	34.99 ± 3.82	−4.59***
Critical thinking of self-confidence	31.99 ± 8.18	32.41 ± 5.52	−1.40
Intellectual curiosity	31.30 ± 6.31	30.83 ± 5.23	1.79
Cognitive maturity	36.28 ± 9.43	36.02 ± 5.95	0.76
Problem-solving ability	89.37 ± 22.41	87.74 ± 14.03	2.04*
Attitude	25.91 ± 7.14	25.14 ± 5.01	2.84*
Methods and strategies	25.73 ± 6.31	25.18 ± 4.33	2.32*
Quality	37.73 ± 10.40	37.42 ± 6.77	0.83
Labor literacy	22.61 ± 8.41	20.15 ± 6.81	7.09***
Labor concept	9.90 ± 3.97	8.79 ± 3.21	6.79***
Labor ability	12.71 ± 4.69	11.36 ± 3.85	6.91***

### 3.4 Linear regression analysis

A multiple linear regression analysis was conducted using labor literacy as the dependent variable and critical thinking disposition and problem-solving ability as the independent variables. The gender variable was dummy-coded before being included in the regression analyses. Then, gender was set as a control variable. The specific results are shown in the table below. It can be seen that: After controlling gender in the study, critical thinking disposition (*β* = 0.245, *p* < 0.001) and problem-solving ability (*β* = 0.288, *p* < 0.001) can significantly positively predict labor literacy, accounting for 20.6% of the total variance, and there is no multicollinearity relationship between the two independent variables (VIF < 5) (Table 6).

**Table 6:** Regression analysis of critical thinking disposition, problem-solving ability and labor literacy.

Dependent variable	Model 1 labor literacy			Model 2 labor literacy		
	*β*	*t*	VIF	*β*	*t*	VIF
Control variable						
Gender	0.047	2.235	1.000	0.031	1.640	1.011
Independent variable						
Critical thinking disposition				0.245	11.699***	1.248
Problem-solving ability				0.288	13.823***	1.240
*R^2^*	0.002			0.206		
*ΔR^2^*	0.002			0.204		
*F*	4.996*			196.229***		

### 3.5 The mediating role of problem-solving ability in critical thinking disposition and labor literacy

To explore the internal mechanism of the influence of critical thinking disposition on labor literacy, in this study, problem-solving ability was introduced as a mediating variable and incorporated into the model. The mediation effect was tested using Model 4 in the SPSS macro program Process 4.1 plugin. The results show that, the upper and lower limits of the 95% bootstrap confidence interval for the mediating effect of critical thinking disposition on labor literacy and problem-solving ability do not include 0 (Table 7).

**Table 7:** Explanatory table for total, direct, and indirect effects.

Effect relationship	Effect	SE	LLCI	ULCI	Ratio
Total effect	0.134	0.007	0.120	0.148	
Direct effect	0.089	0.008	0.074	0.104	66.42%
Indirect effect	0.045	0.005	0.035	0.055	33.58%

SE, standard error; LLCI, lower limit confidence interval; ULCI, upper limit confidence interval.

**Figure 2:**
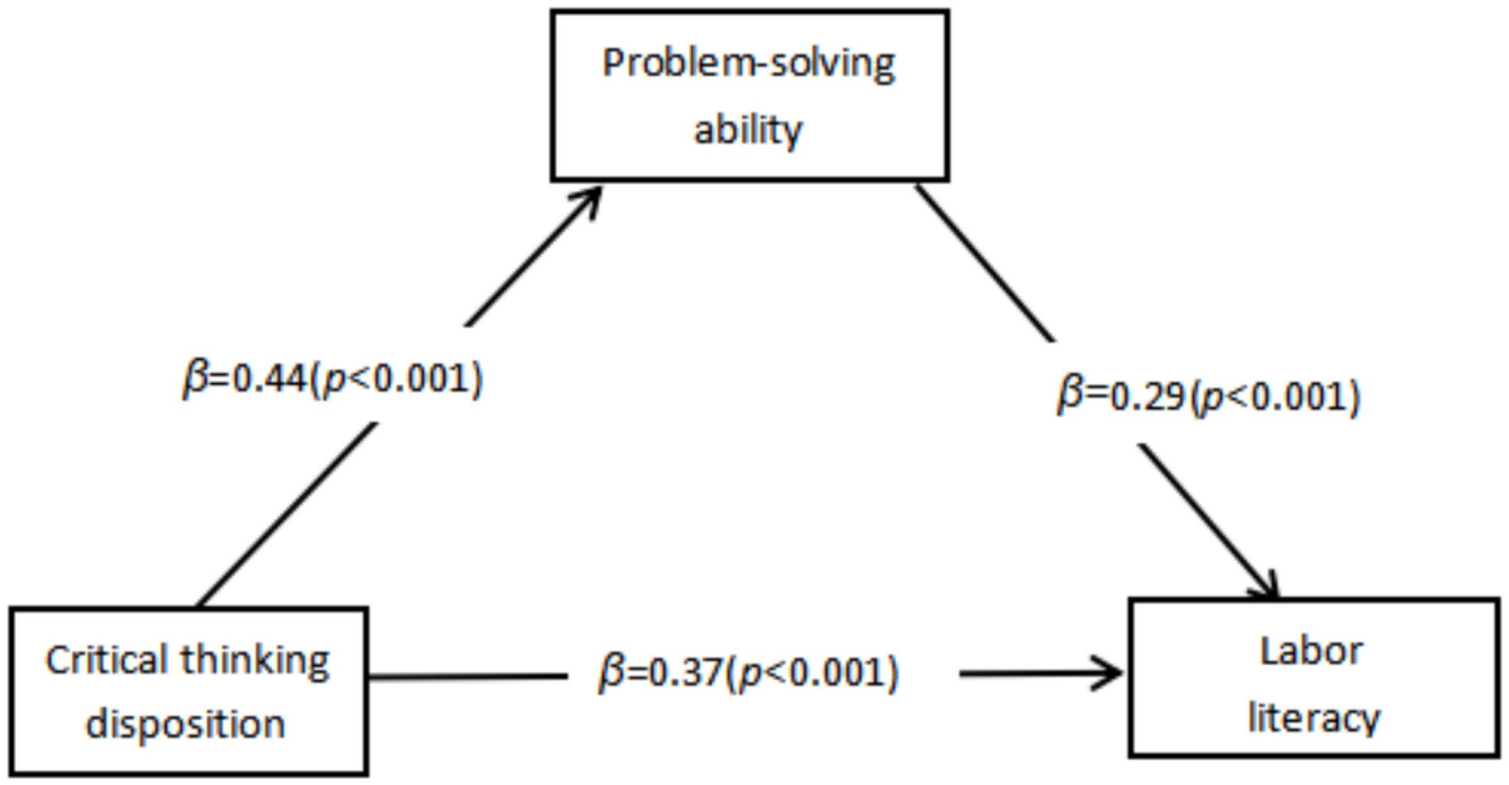
Mediating effect model.

This indicates that the critical thinking disposition not only has a direct effect on labor literacy, but also can play an indirect mediating role in labor literacy through the variable of problem-solving ability. The specific test results are shown in the table below. The direct effect (0.089) and the indirect effect (0.045) respectively account for 66.42 and 33.58% of the total effect (0.134). This suggests that approximately 33.58% of the positive impact of critical thinking disposition on labor literacy is achieved by improving the problem-solving ability of teenagers. The mediating effect model is presented in Figure 2 (Using standardized coefficients).

## 4 Discussion

This study analyzed the differences in critical thinking disposition, problem-solving abilities, and labor literacy among individuals in terms of demographic variables. It further revealed the current development status of these three aspects and their correlations with educational background and social cultural factors, providing a foundation for the subsequent in-depth exploration of the influencing mechanisms.

### 4.1 Analysis of critical thinking disposition, problem-solving ability and labor literacy on demographic variables

The research results show that the scores of Chinese teenagers in critical thinking disposition (*M* = 236.76), problem-solving ability (*M* = 88.95), and labor literacy (*M* = 21.97) are all at a moderately low level. This phenomenon indicates that there is still considerable room for improvement in the development of these key competencies among Chinese teenagers. This is related to the current educational practice that often focuses on knowledge transmission while relatively neglecting thinking training and labor experience. It is also consistent with the conclusion in the PISA 2022 Creativity Thinking Assessment Report released by the Organization for Economic Cooperation and Development (2024), which points out that some education systems still face challenges in cultivating students’ creative thinking. This indicates that merely including “all-round development” or “core literacy” in the guiding documents is insufficient. Systemic reforms such as curriculum standards, teacher training, and evaluation systems must be carried out to transform the literacy goals into practical teaching practices, truly promoting educational transformation.

In terms of gender, the research found that the overall scores of boys in critical thinking disposition and labor literacy were significantly higher than those of girls. In terms of critical thinking disposition, boys significantly outperformed girls in the dimensions of Open-mindedness, Analysis ability, Intellectual Curiosity, and Cognitive maturity, while in the dimension of Critical thinking of self-confidence, girls showed a significant advantage. In terms of labor literacy, boys scored significantly higher than girls in the Labor concept dimension. The differences in labor literacy may be related to the social learning theory (Bandura, 1977) and the perspective of gender socialization: In traditional gender roles, men are assigned the responsibility of “providing for the family”, and society has higher expectations for men in terms of labor, which may prompt boys to receive more labor training and recognition in daily life, thereby improving their labor literacy performance and related concepts. The gender differences in critical thinking disposition may partly stem from the differences in cognitive style preferences shaped by social culture (Sternberg, 1997). For example, in the classic dimension of “field dependence - field independence”, studies show that the male group is usually stronger in field independence, that is, they are better at separating problems from the background and conducting abstract analysis (Witkin et al., 1977), and thiscognitive style precisely aligns with the core skills required for critical thinking such as analysis. The advantage of girls in critical thinking of self-confidence may be related to their experiences in communication and emotional expression, which helps to form a positive evaluation of their own judgment and expression abilities. This indicates that literacy cultivation should have a gender perspective. On one hand, through curriculum and activity design, we should actively encourage girls to develop confidence and abilities in analytical and systematic thinking and other field-independent cognitive styles; on the other hand, we should guide boys in the development of social emotional abilities such as collaboration and expression.

In terms of place of residence, there are no significant differences in the three variables between urban and rural students. This may reflect the effectiveness of the integrated development of education in urban and rural areas in China. The policy of balancing educational resources has to some extent narrowed the gap in the development of thinking and qualities between urban and rural students. In the future, further exploration can be conducted on which specific policy interventions (such as teacher exchanges, resource transfer, and curriculum collaboration) are the most effective, thereby providing a “Chinese case” for promoting educational equity globally.

In terms of academic stage differences, the research shows that junior high school students scored significantly higher than senior high school students in the total score of problem-solving ability, as well as in the dimensions of attitude, methods and strategies, and in the total score of labor literacy, as well as in the dimension of labor concepts and labor abilities. However, in the systematic thinking dimension of critical thinking disposition, junior high school students scored significantly lower than senior high school students. This may be related to the different curriculum structures and goals of education at different stages in China. The curriculum setting in the junior high school stage is relatively diverse, and there are certain social practice courses retained, allowing students to have more opportunities to participate in practical and foundational problem-solving and labor practices, which is conducive to the development of labor literacy. In the senior high school stage, due to the increased pressure of academic advancement, the educational focus shifts toward academic knowledge, and practical courses may be compressed, and students may have fewer opportunities to participate in real-life practical activities. This leads to a slow development of related abilities and qualities. Meanwhile, senior high school students’ advantage in the systematic thinking dimension may reflect the natural growth of their ability in logical integration and systematic analysis as they develop cognitively and deepen their subject knowledge, demonstrating the promoting effect of academic training on this thinking trait. This reveals the problem between the “exam-oriented” education system and “quality development” in the current education system, and poses a practical challenge for integrating labor education and thinking cultivation in the senior high school stage. It indicates that the comprehensive reform in the senior high school stage,especially the effective implementation of the college entrance examination reform and the comprehensive quality evaluation, has become a key institutional solution to this contradiction and to ensuring the continuity of quality cultivation.

### 4.2 The direct impact of critical thinking disposition on the labor literacy of teenagers

The research results indicate that the critical thinking disposition among teenagers has a significant positive predictive effect on their labor literacy, and Hypothesis H2 has been verified. That is, teenagers with a higher critical thinking disposition exhibit higher levels in both labor concepts and labor abilities. This finding confirms the core value of critical thinking in cultivating labor literacy. Renowned educational scholar Zhao Yong emphasizes that in the era of globalization and intelligence, the core task of education should shift from knowledge transmission to cultivating critical thinking, creativity, and entrepreneurial spirit, which are the fundamental aspects for students to create value and develop in complex social and professional environments in the future (Zhao, 2012, 2018). The conclusion of this study is highly consistent with this: Critical thinking not only guides teenagers to actively think about the meaning of labor (at the conceptual level), but also endows them with the ability to analyze and solve problems in real labor situations (at the ability level). Existing research has found that people with high levels of critical thinking ability not only have stronger entrepreneurial capabilities but also tend to have higher income levels (Martinez et al., 2007), and this thinking ability is regarded as an important skill connecting academic success and coping with complex social and professional environments (Ran, 2025). These evidences confirm that critical thinking is the core cognitive engine driving creative labor and occupational adaptability. Therefore, the conclusion of this study deepens the theoretical connotation of labor education, indicating that labor education should not only remain at the level of skill transmission and behavior training, but also should attach importance to the cultivation of thinking qualities as its cognitive foundation. Labor education activities should incorporate the goal of thinking training, for example, issuing learning tasks based on actual situations, organizing scheme discussions before the start of the tasks, guiding students to analyze the conditions and feasibility of the tasks; setting problem-solving sections during the tasks to encourage them to identify and solve difficulties in practical operations;and conducting multi-angle summaries after the tasks to guide students to reflect from aspects such as process,cooperation,and results. Through such design, thinking training can naturally run through the entire process of labor practice.

### 4.3 The mediating role of problem-solving ability

The results of the mediation effect analysis in this study indicate that the critical thinking disposition of adolescents not only directly positively predicts their labor literacy level, but also indirectly affects labor literacy through the mediating role of problem-solving ability, thereby verifying Hypothesis H4. Based on the framework of cognitive development theory (Piaget, 1970) and social-cultural theory (Vygotsky, 1978), this can be further explained: Critical thinking, as a high-level cognitive ability, helps adolescents to self-regulate and construct meaning in labor practice, while problem-solving ability acts as a key bridge for converting internal cognition into explicit labor behavior. Specifically, the critical thinking disposition enables adolescents to more actively identify problems, analyze information, and evaluate solutions in real labor situations, thereby providing a cognitive foundation for effective problem-solving (Facione, 1990). The problem-solving ability plays a key role in this process, prompting individuals to better master knowledge through autonomous learning (Liu et al., 2025). This mechanism also echoes the educational philosophy of “learning by doing” (Dewey, 1986), indicating that thinking training must be combined with actual problem-solving to achieve the internalization and development of labor literacy. In labor education practice, educators should attach importance to the cultivation of critical thinking and integrate labor tasks with real problem scenarios. For example, through project-based learning, situational simulation, etc., teaching strategies can be used to guide students to make decisions and reflect in complex and open labor scenarios, thereby exercising their comprehensive problem-solving abilities (Wagner, 2008). This not only helps with the acquisition of labor knowledge and skills, but also promotes the formation of labor qualities such as responsibility and cooperation. In conclusion, this study clarifies the psychological path through which the critical thinking disposition influences labor literacy via problem-solving ability from an empirical perspective, providing theoretical basis and practical implications for integrating thinking training and actual problem-solving in labor education. Further deepening the theoretical construction and empirical exploration in this field will help promote the implementation of labor education toward a more systematic and scientific path.

### 4.4 Limitations of the research

This study explored and verified the mediating role of problem-solving ability between the critical thinking disposition and labor literacy of adolescents, providing preliminary empirical evidence for understanding the relationship between the two. However, this study still has several limitations, and future research can further deepen and expand on this basis.

In terms of sample representativeness, this study has certain limitations. The data collection mainly came from two provinces in China. Although the sample size meets the basic statistical requirements, there may be imbalances in terms of regional distribution, school types, etc. China has a vast territory, and there are differences in economic and social development levels, resource allocation of education, and cultural environment in different regions. These factors may systematically affect the development of teenagers’ thinking patterns and labor skills, thereby limiting the generalization of the research conclusions. Future research can adopt national stratified sampling to include more diversified samples from different regions, school types, and social cultural backgrounds to test the applicability of the current model.

In terms of measurement methods, this study mainly relies on self-report scales, which carry a certain risk of common method bias. Although the scales used have good reliability and validity, the measurement of variables is based on students’ self-reports, which may be influenced by social approval, self-perception biases, or subjective understanding differences. Especially for variables such as problem-solving ability, it might be more suitable to conduct objective assessment through behavioral measurement. Using only self-report scales may not fully capture their actual performance, thereby introducing additional subjectivity. Future research can adopt multi-method measurement strategies, such as combining teacher evaluations, behavioral observation records, or standardized situational problem-solving tasks, to assess relevant variables more comprehensively and objectively, and enhance the robustness of the research results.

In terms of research design, this study revealed the correlations between variables and the mediating paths, but the data were collected at a single time point, making it impossible to strictly determine the causal direction of the changes in critical thinking disposition, problem-solving abilities, and labor literacy over time. For instance, an improvement in labor literacy might also promote the development of critical thinking in a reverse manner. Future research could adopt a longitudinal tracking design, collecting data at multiple time points, or attempt to design educational intervention experiments (such as conducting critical thinking training courses), by comparing the changes before and after the intervention in the experimental group and the control group, to provide more solid evidence for the causal relationships between the variables.

## 5 Conclusion

This study has demonstrated the relationship between the critical thinking disposition and problem-solving ability of adolescents and their labor literacy. It shows that adolescents’ critical thinking can positively influence labor literacy, and their problem-solving abilities can also indirectly positively affect labor literacy. Future educational practices should establish an integrated system for cultivating competencies.

Therefore, based on the research results, schools should adopt various teaching methods to enhance adolescents’ critical thinking, problem-solving abilities, and labor literacy. The research suggests:

(1) In terms of curriculum design, schools can offer interdisciplinary activity programs that are integrated with the school-based curriculum. This approach is more in line with students’ real-life situations and encourages students to analyze problems from multiple perspectives, explore various solutions, and verify the feasibility and effectiveness of the solutions, thereby generating creative products. For example, specific problems based on students’ actual situations can be identified, and multiple subjects can be integrated to enable students to develop their thinking and qualities in the process of solving real problems.
(2) In terms of teaching strategies, heuristic teaching can be adopted. By analyzing, solving and reflecting on problems step by step, students’ thinking can be exercised. Conflict situations can also be created to deepen thinking training through debates and other means.
(3) In terms of the evaluation mechanism, based on students’ “problem-solving ability,” grade assessment is conducted through practical achievements, rewards are awarded and incorporated into the comprehensive quality evaluation to discover students’ specialties. During the college entrance examination, the comprehensive quality evaluation file can be included in the scope of investigation for selective admission.

## Data Availability

The raw data supporting the conclusions of this article will be made available by the authors, without undue reservation.
